# Crosstalk Between Intestinal Serotonergic System and Pattern Recognition Receptors on the Microbiota–Gut–Brain Axis

**DOI:** 10.3389/fendo.2021.748254

**Published:** 2021-11-08

**Authors:** Elena Layunta, Berta Buey, Jose Emilio Mesonero, Eva Latorre

**Affiliations:** ^1^ Institute of Biomedicine, Department of Medical Biochemistry and Cell Biology, University of Gothenburg, Gothenburg, Sweden; ^2^ Instituto de Investigación Sanitaria de Aragón (IIS Aragón), Zaragoza, Spain; ^3^ Departamento de Farmacología, Fisiología y Medicina Legal y Forense, Universidad de Zaragoza, Zaragoza, Spain; ^4^ Instituto Agroalimentario de Aragón—IA2 (Universidad de Zaragoza–CITA), Zaragoza, Spain; ^5^ Departamento de Bioquímica y Biología Molecular y Celular, Universidad de Zaragoza, Zaragoza, Spain

**Keywords:** serotonin, 5-HT, tryptophan, microorganisms, PRRs, TLRs, NLRs

## Abstract

Disruption of the microbiota–gut–brain axis results in a wide range of pathologies that are affected, from the brain to the intestine. Gut hormones released by enteroendocrine cells to the gastrointestinal (GI) tract are important signaling molecules within this axis. In the search for the language that allows microbiota to communicate with the gut and the brain, serotonin seems to be the most important mediator. In recent years, serotonin has emerged as a key neurotransmitter in the gut–brain axis because it largely contributes to both GI and brain physiology. In addition, intestinal microbiota are crucial in serotonin signaling, which gives more relevance to the role of the serotonin as an important mediator in microbiota–host interactions. Despite the numerous investigations focused on the gut–brain axis and the pathologies associated, little is known regarding how serotonin can mediate in the microbiota–gut–brain axis. In this review, we will mainly discuss serotonergic system modulation by microbiota as a pathway of communication between intestinal microbes and the body on the microbiota–gut–brain axis, and we explore novel therapeutic approaches for GI diseases and mental disorders.

## 1 Introduction

The gastrointestinal (GI) tract is one of the major defensive organs in individuals because it is continuously exposed to the external environment. In this context, microbial colonization of the intestine during infancy is a major moment for the development of not only the GI tract (1) but also the brain (2) and the immune system (3). In the last years, numerous researchers have focused their efforts on understanding how intestinal microbiota have the ability to affect the brain and behavior, which has not yet been completely clarified. In this context, the neurotransmitter serotonin (5-hidroxytriptamine, 5-HT) could be the key to resolving this mystery.

The gut–brain axis is a bidirectional crosstalk between the central nervous system (CNS) and the gut. Recently, given the important role in the regulation of gut functions, microbiota are included in the axis. Then, the microbiota–gut–brain axis resides in a coordinated network composed of the CNS, enteric nervous system (ENS), hypothalamic–pituitary–adrenal axis, gut, and microbiota. Both clinical and experimental data suggest that intestinal microbiota play a crucial role in the axis, interacting not only locally with intestinal cells and the ENS but also directly with the CNS through neuroendocrine and metabolic pathways. In fact, germ-free mice studies have proven that the absence of microbial colonization leads to defects in neuron maturation at both CNS and ENS levels, altered expression of neurotransmitters, and gut sensory and motor dysfunctions (4). Intestinal microbiota dysbiosis has been extensively studied as one of the most important factors in the pathogenesis of inflammatory bowel diseases (IBDs) (5), including Crohn’s disease (CD) and ulcerative colitis (UC). In this context, several studies have described that intestinal serotonin may shape the microbiota composition that protects against the development of IBDs (6), suggesting the critical relation between the intestinal microbiota and serotonergic system in GI pathologies. However, the role of the microbiota–serotonin interaction would not be limited locally to the gut but also to the CNS. Germ-free mice studies have reported the importance of the microbiota control of the serotonergic system in the CNS (7) or how specific intestinal microorganisms, such as *Akkermansia muciniphila*, can increase serotonin production in the hippocampus (8). In this context, recent studies have described the involvement of microbiota in serotonin signaling in CNS disorders such as Alzheimer’s or schizophrenia (9).

Serotonin is a key neurotransmitter, which substantially coordinates the GI physiology and owns critical central functions. Interestingly, serotonin is involved in each component of the microbiota–gut–brain axis, acting as an ideal language for the crosstalk. Microbiota regulate the tryptophan metabolism involved in serotonin production, serotonin acts as a key neurotransmitter in the CNS and ENS, and serotonin receptors play a pivotal role in the hypothalamic–pituitary–adrenal axis.

Here, we highlight recent findings into how microbiota regulate the intestinal and central serotonergic systems, as well as novel clinical approaches to address GI pathologies and brain disorders through the microbiota–gut–brain axis.

## 2 Serotonergic System

**Figure 1:**
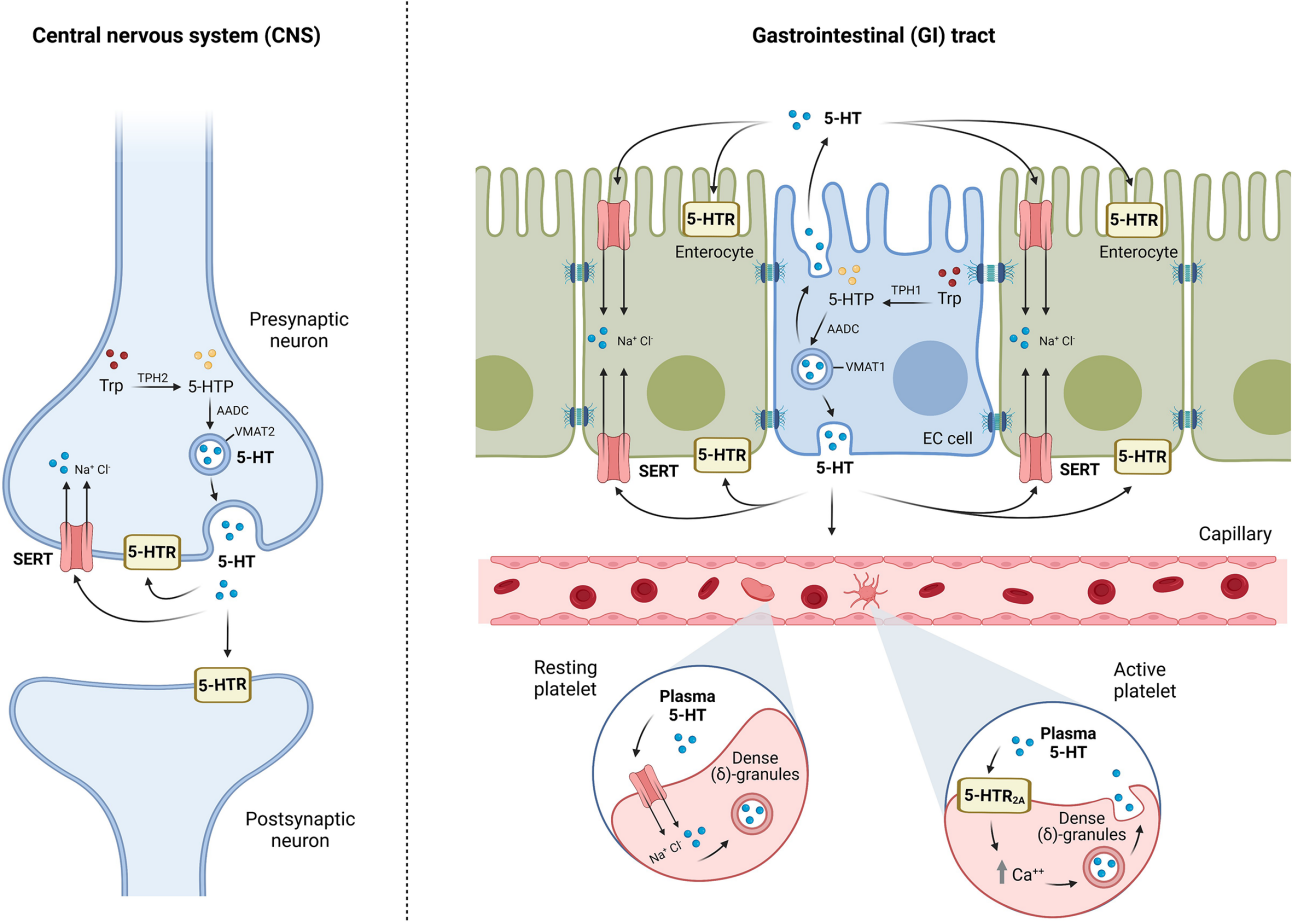
Schematic representation of brain and intestinal serotonergic systems: “ON/OFF” and signaling mechanisms. “ON” mechanism refers to the synthesis of 5-HT by enterochromaffin cells (EC) in the gut and serotonergic neurons both in the gut and in the central nervous system (CNS). Tryptophan (Trp) is catalyzed by the enzyme tryptophan hydroxylase (TPH), TPH1 in EC cells, and TPH2 in neurons, to synthesize 5-hydroxytryptophan (5-HTP), which is converted to 5-HT by aromatic amino acid decarboxylase (AADC). 5-HT is stored into vesicles through the vesicular monoamine transporter VMAT (VMAT1 in EC cells, and VMAT2 in neurons) and finally released into the extracellular space. 5-HT can bind to different serotonin receptors (5-HTR) or uptake into neurons, enterocytes, or platelets by the serotonin transporter (SERT), ending 5-HT effects (“OFF” mechanism). 5-HT is mostly stored in the dense (δ)-granules of platelets; however, the binding of plasma 5-HT to the platelet surface receptor 5-HT_2A_ initiates the mobilization of intracellular calcium stores for platelet activation, which promotes platelet degranulation, resulting in 5-HT release. Serotonin exerts its effects by signaling mechanisms through the 5-HT receptors located in postsynaptic and presynaptic neurons at CNS and intestinal serotonergic neurons, and in different cell types of gastrointestinal (GI) tract, but also in other systems such as the cardiovascular or immune system.

In 1940, Vittorio Erspamer discovered serotonin (5-hydroxytryptamine, 5-HT) in the GI tract in rabbits (10) and it was later discovered in the CNS (11). There are two main serotonergic systems: the central serotonergic system located in the brain and the intestinal serotonergic system in the gut. Both share the same principles of synthesis (“ON mechanism”), internalization and degradation (“OFF mechanism”), and 5-HT signaling through its specific receptors (**Figure 1**).

- The “ON” mechanism is constituted in the gut by enterochromaffin cells and serotonergic neurons of the ENS, while in the CNS, 5-HT is produced only by serotonergic neurons. The primary source of 5-HT is the amino acid L-tryptophan that is catalyzed by the rate-limiting enzyme tryptophan hydroxylase (TPH) to synthesize 5-hydroxytryptophan (5-HTP), which then is converted into serotonin by aromatic amino acid decarboxylase (AAAD) (12). TPH reaction is a limitative step in the production of 5-HTP and, subsequently, serotonin. It has been described in two isoforms of TPH: TPH1 expressed in enterochromaffin cells and TPH2 in serotonergic neurons from both the ENS and CNS (13).
- The “OFF” mechanism in the gut is formed by enterocytes because these intestinal epithelial cells (IECs) internalize 5-HT from the extracellular compartment to the cytoplasm by means of the serotonin transporter (SERT) from the apical and the basolateral membranes. At the CNS level, the “OFF” mechanism is formed by the same serotonergic neurons that synthesize 5-HT because SERT is expressed at terminals and varicosities of serotonergic neurons (14). SERT is a transmembrane protein grouped in the solute carrier transporters of the SLC6 family that uptakes 5-HT from the extracellular space for subsequent catabolization, reuse, or storage, ending 5-HT effects. SERT is a classic secondary active transporter to which 5-HT binds together with a Na^+^ and a Cl^-^. Once extracellular serotonin is attached to SERT together with Na^+^ and Cl^-^, SERT undergoes a conformational change that allows SERT translocation with the release of 5-HT, Na^+^, and Cl^-^ into the cytoplasm of the cell. Once 5-HT is transported inside the cell, intracellular K^+^ binds to SERT and is reoriented toward the extracellular direction, where K^+^ is released and the uptake of 5-HT continues. Then, SERT is not only a key component for the regulation of 5-HT levels, but also an important ion transporter (15).
- 5-HT signaling is mediated by specific serotonin receptors that trigger intracellular 5-HT effects (**Table 1**). Scientific community studies on serotonin receptors have recently described a detailed work that classifies the 18 receptors grouped into seven families (5-HT1 to 5-HT7), which are widely expressed not only in the CNS and the GI tract but also in other systems such as the cardiovascular or immune system (79). As a short summary, the serotonin receptor family consists of G-protein-coupled receptors, with the exception of the 5-HT_3_ receptor family (80). 5-HT_1_ includes five subtypes: 5-HT_1A_, 5-HT_1B_, 5-HT_1D_, 5-HT_1E_, and 5-HT_1F_. They are fundamentally involved in CNS disorders such as anxiety. In the case of the GI tract, the 5-HT_1_ family is mainly expressed in neurons of the gut submucosa and the myenteric plexus, so their main function is the modulation of GI motility (18). The 5-HT_2_ family involves 5-HT_2A_, 5-HT_2B_, and 5-HT_2C_. 5-HT_2A_ and 5-HT_2B_ are expressed in myenteric neurons and neurons from the submucosal plexus in the GI tract, as well as in enterocytes and smooth muscle cells in the gut (36). Thus, the effect of these receptors is mainly in the GI tract through the regulation of GI motility (81). However, these receptors are expressed in the brain, where they may control central processes such as memory and cognition (82) or be implicated in CNS disorders such as depression (83). 5-HT_2C_ is mainly expressed in the CNS and is involved in several central processes such as the limbic system and motor behavior (38). The 5-HT_3_ family includes five receptors (5-HT_3A-D_) and works as an ion channel similar to GABA receptors. 5-HT_3_ receptors are expressed in both the CNS and the GI tract and are involved in several GI processes such as intestinal motility (84), absorption and secretion (85), and even 5-HT release from enterochromaffin cells (86); in the brain, 5-HT_3_ receptors are related with cognition (87). In this context, 5-HT_3_ family dysfunction has been involved in a broad range of pathologies from brain disorders, including psychosis, anxiety, and eating disorders (43), to GI pathologies such as IBDs (88). 5-HT_4_ receptors are mainly expressed in the gut and participate in intestinal secretion (63) and motility (53). The 5-HT_5_ receptor is the least known from the serotonergic system as some researchers have referred to it for two decades as “the orphan serotonin receptor” (89). Despite the limited information about this 5-HT receptor, the scientific community has established two subtypes expressed exclusively in the nervous system: 5-HT_5A_ and 5-HT_5B_ (90). These receptors may be involved in several processes, including memory (65) or pain (67). 5-HT_6_ receptors, such as 5-HT_5_ receptors, have also been poorly studied. Previous studies in mice have highlighted that it may be important in the GI physiology; however, its importance is not clear (91). At the CNS level, 5-HT_6_ is involved in mental disorders, such as psychosis, and in cognition and learning (70). Finally, the 5-HT_7_ receptor is mainly expressed in the brain but is also located in peripheral organs such as the GI tract (73). The 5-HT_7_ receptor is involved in circadian rhythm (78), and its dysfunction is important in the onset of depression (92). In the GI tract, 5-HT_7_ modulates SERT activity (75) and intestinal motility (77).

**Table 1:** 5-HT receptors.

Receptor	Subtypes	Location	Mechanism	Gastrointestinal Function	CNS Function
5-HT1	5-HT_1A_ 5-HT_1B_ 5-HT_1D_ 5-HT_1E_ 5-HT_1F_ 5-HT_1P_	CNS: Hippocampus, neocortex, raphe nuclei, cerebellum, and basal ganglia (16) GI: Neurons of the gut submucosa and the myenteric plexus (17) Other locations: Lymph nodes, thymus and spleen, activated T cells, but not in resting T cells (18, 19)	G-protein-coupled receptor for 5-HT that inhibits adenylate cyclase (20)	Modulation of the intestinal motility (21). Modulation of gastric motility and sensitivity (22) Degranulation of enteric mast cells and release of mediators (23) Inflammation (24)	Addiction (25) Behavior (26, 27) Appetite (28) Memory (29) Sleep (30)
5-HT2	5-HT_2A_ 5-HT_2B_ 5-HT_2C_	CNS: Cerebellum, lateral septum, hypothalamus, hippocampus, middle part of the amygdala, and cortex (31) GI: Myenteric neurons and neurons from the submucosal plexus at the GI tract, in enterocytes and smooth muscle cell (32) Other locations: Heart and kidney (33)	G-protein-coupled receptor for 5-HT that activates phospholipase C (20)	Modulation of the intestinal motility (34) Enterocyte secretion (35) Development of enteric neurons (36)	Behavior (37) Memory and cognition (38 Limbic system or motor behavior (39)
5-HT3	5-HT_3A_ 5-HT_3B_ 5-HT_3C_ 5-HT_3D_	CNS: Hippocampus, dorsal motor nucleus of the solitary tract and area postrema, olfactory bulb, the trochlear nerve nucleus, the dorsal tegmental region, the facial nerve nucleus, the nucleus of the spinal tract of the trigeminal nerve, and the spinal cord dorsal horn (40) GI: Enteric neurons, smooth muscle cells, vagal and spinal primary afferent neurons, and in the spinal cord (41) Other locations: Dorsal root ganglia (40)	Ligand-gated ion channels (LGIC) that mediates neuronal depolarization and excitation (42)	Intestinal motility (43) Inflammation (44) Colonic secretion (45) Intestinal pain and sensitivity (46)	Release control of other neurotransmitters: dopamine, GABA or acetylcholine among others (47). Regulation of emesis (48) Neurodevelopment (49) Anxiety (50)
5-HT4		CNS: Cortical areas, hippocampus, olfactory tubercles (51) GI: Enteric neurons and smooth muscle cells (52) Other locations: Heart muscle and pituitary gland (Protein Atlas)	G-protein-coupled receptor for 5-HT that promote cyclic AMP formation (53)	Motility (54) Absorption (55) Intestinal sensitivity (56)	Memory and cognition (57, 58) Behavior (59) Feeding (60)
5-HT5	5-HT_5A_ 5-HT_5B_	CNS: Cerebral cortex, hippocampus and cerebellum (61)	G-protein-coupled receptor for 5-HT that regulates adenylate cyclase (62)	Intestinal secretion (63)	Behavior (64) Memory and cognition (64, 65) Sensory perception and neuroendocrine function (66) Pain (67)
5-HT6		CNS: Olfactory tubercle, cerebral cortex (frontal and entorhinal regions), hippocampus, and cerebellum among others (68)	G-protein-coupled receptor for 5-HT that regulates adenylate cyclase (69)		Learning and cognition (70) Release control of other neurotransmitters (71) Motor control (72)
5-HT7		CNS: Thalamus, hypothalamus, limbic, and cortical regions (73) GI: Gut-associated neurons, but also in enterocyte-like and immune cells in lymphatic tissues (74) Other locations: Spleen, kidney, heart, coronary artery immune cells (73)	G-protein-coupled receptor for 5-HT that regulates adenylate cyclase (74)	SERT activity modulation (75) Intestinal motility (76) Inflammation (74)	Inflammation and repair (77) Circadian rhythm (78)

Localization, mechanism, and gastrointestinal (GI) and central nervous system (CNS) functions.

## 3 Microbial Pattern Recognition Receptors: Effects on Serotonergic System

Defense mechanisms in the intestine are widely developed because external agents are in continuous contact with the intestinal epithelium. Innate immunity, throughout several detectors called pattern recognition receptors (PRRs), detects external factors, triggering either tolerant or defense responses to beneficial or pathogenic molecules, respectively. The most important and studied PRRs are microbial detectors: toll-like receptors (TLRs) and nucleotide oligomerization domain (NOD)-like receptors (NLRs) (**Table 2**). TLRs are transmembrane glycoproteins, whereas NLRs are cytosolic receptors. Until now, 11 different TLRs have been identified in humans (TLR1–TLR11) and expressed in both the endosomal membrane (TLR3, 7, 8, and 9) and cell membrane (TLR1, 2, 4, 5, 6, 9, and 10) (107). Regarding NLRs, 22 receptors have been discovered until now, which can be classified into five groups depending on their structure: NLRA, NLRB, NLRC, NLRP, and NLRX (141).

**Table 2:** Pattern recognition receptors: TLRs and NLRs.

Receptor	Cellular location	Tissue location	Intracellular Mechanism	MAMPs	DAMPs
TLR2	Plasma membrane	CNS: Microglia, astrocytes and oligodendrocytes (93) GI: Mononuclear cells of the lamina propria, goblet cells, enterocytes, and neurons from the ENS (94, 95)	TLR2 forms heterodimers with TLR1 and TLR6 to detect most of its specific ligands. Then, it generally triggers a MyD88-dependent signaling pathway to promote the translocation of nuclear factor-B that regulate the synthesis of inflammatory factors (96)	Molecules with diacyl and triacylglycerol moieties, proteins, and polysaccharides (96)	HSP60 and HSP70 (97) HMGB1 (98) Gp96 (99)
TLR3	Endosomal membrane	CNS: Astrocytes, oligodendrocytes, and microglia cells (93) GI: Immune cells of lamina propria and IECs including goblet cells and enterocytes (100) and in neurons from ENS (101).	TLR3 activation triggers TRIF/TICAM1 intracellular signaling that ends in the NF-kappa-B activation with IRF3 nuclear translocation and the synthesis and release of inflammatory factors (102)	Double-stranded (ds) RNA (103)	Endogenous mRNA from inflammation (104)
TLR4	Plasma membrane	CNS: Microglia cells (105), astrocytes (106) GI: Immune cells of lamina propria, in the apical membrane of IECs in small intestine and in the basolateral membrane in the colon (107). Moreover, it can be found in neurons from ENS (101)	TLR4 can trigger a Myd88-dependent signaling pathway and a Myd88-independent intracellular signaling pathway driven by TRIF to promote the translocation of nuclear factor-B that regulate the synthesis of inflammatory factors (108)	Lipopolysaccharide (109)	HMGB1 (110) Fibrinogen (111) HSP60, HSP72, SP22 (112) Lactoferrin (113)
TLR5	Plasma membrane	CNS: Microglia cells (93) GI: Basolateral side of IECs at the colon, at Paneth cells at the small intestine while in small intestine its expression is restricted to Paneth cells.	TLR5 activation triggers MYD88 and TRIF intracellular signaling that leads to the translocation of NF-kappa-B and inflammatory response (114, 115)	Flagellin (116)	HMGB1 (117) Hyaluronan (118)
TLR7	Endosomal membrane	CNS: Microglia cells (93) GI: IECs, plasmacytoid dendritic cells (pDCs), B cells at the lamina propria (119), and in the myenteric and submucous plexuses of murine intestine and human ileum (101).	TLR7 activation triggers MYD88 intracellular pathway signaling that leads to the activation NF-kappa-B and IRF7 to promote the synthesis of inflammatory factors (120)	ssRNA (121)	Guanosine and short O(R)Ns from RNA degradation (122) ssRNA (123)
TLR8	Endosomal membrane	CNS: Microglia cells (93) GI: Macrophages and monocyte-derived DCs at lamina propria (121)	TLR8 activation recruits MYD88 intracellular pathway signaling that activates NF-kappa-B and IRF7 to promote the synthesis of inflammatory factors (124)	ssRNA (121)	ssRNA (123) Uridine and short ORNs from RNA degradation (122)
TLR9	Endosomal membrane/Plasma membrane	CNS: Microglia, neurons, and astrocytes (125) GI: Immune cells from lamina propria in GI epithelial cells (119)	TLR9 activation induce MYD88 and TRAF intracellular pathway downstream that leads into the activation of NF-kappa-B (126)	Unmethylated cytidine-phosphate-guanosine (CpG) dinucleotides (127)	IgG–chromatin complexes (128) Host DNA degradation (129)
TLR10	Plasma membrane	CNS: Microglia (130) GI: B-cells (131) and IECs (132)	TLR10 may trigger intracellular responses MyD88-dependent and MYD88-independent downstream signaling (132)	Unknown MAMPs Candidates as a TLR10 ligand: diacylated lipopeptides (133) and lipopolysaccharide (133)	Unknown DAMPs
NOD1	Intracellular compartment	CNS: Microglia (134), neurons, and astrocytes at prefrontal cortex, hippocampus, and cerebellum (135) GI: IECs and in the immune cells from lamina propria (136)	NOD1 recruits RIPK2, which promotes interactions with TRAF, and activates the expression NF-κB and MAPK involved in inflammatory responses (137)	κ-d-glutamyl-meso-diaminopimelic acid (136)	Endoplasmic reticulum stress molecules (138) Calcium (138)
NOD2	Intracellular compartment	CNS: Microglia (134) GI: Monocytes, dendritic cells, epithelial cells, Paneth cells, and intestinal stem cells (139)	NOD2 recruits RIPK2, which promotes interactions with TRAF, and activates the expression NF-κB and MAPK involved in inflammatory responses (137)	Muramyl dipeptide (140)	Endoplasmic reticulum stress molecules (138) Calcium (138)

Indication of intracellular location, expression at the central nervous system (CNS) and gastrointestinal tract (GI), main intracellular mechanism, main microbial-associated molecular patterns (MAMPs), and main damage-associated molecular patterns (DAMPs).

PRRs are widely expressed in immune cells (phagocytes, neutrophils, macrophages, or lymphocytes) and nonimmune ones, such as IECs in the GI tract, as well as microglia cells, neurons, or astrocytes in the CNS. PRRs trigger defense-related responses by the detection of specific microbial-associated molecular patterns from microorganisms (MAMPs) or damage-associated molecular patterns (DAMPs) from tissue injury, so we can consider the PRRs the caretakers of our body.

PRRs functioning in IECs are focused on the protection of the intestinal epithelium from potential harmful agents. Thus, and through PRR signaling, the intestine continuously develops the status of physiological inflammation to prevent possible damage and maintain intestinal homeostasis (142). In the brain, the main role of the PRRs is to detect dangerous molecules that can injure the tissue and trigger repair mechanisms. The brain is protected by the skull, the fluid cerebrospinal, the meninges, and the blood–brain barrier (BBB), which isolates the CNS from the general circulation. However, under pathological conditions, harmful microorganisms can breach the BBB and access the CNS, where the PRRs can trigger defense mechanisms to eliminate the pathogen and to repair the tissue (143).

PRRs are widely expressed along the GI tract, which differs dramatically between the small intestine and colon (122). From all of them, TLR2, TLR3, TLR4, TLR5, and TLR9 seem to be critical in microbial detection and damage repair in the intestine. In the brain, the most studied PRRs, in relation with brain injury and pathogen infection, are TLR2, TLR3, TLR4, and TLR9. However, the scientific community does not discard the relevant importance of other TLRs in this location because they are expressed in several cells from the CNS (125). PRRs influence the serotonergic system activity and expression (**Table 3**).

**Table 3:** Pattern recognition receptors on serotonergic system.

Pattern Recognition Receptor	Effects on serotonergic system	Model	References
TLR2 activation	Decreased SERT	IEC model	(144)
	Upregulated TPH1 expression and 5-HT production	GF mice	(145)
TLR3 activation	Inhibited SERT	IEC model	(146)
	Increased SERT activity	Astrocytes	(147)
TLR4 activation	Inhibited SERT activity	IEC model	(148)
	Enhanced cortical SERT activity	Wistar rats	(149)
TLR7/8 activation	Inhibited 5-HT_2B_ signaling	Dendritic cells	(150)
TLR10 activation	Regulation of SERT activity	IEC model	(131)
NOD1 activation	Decreased SERT	IEC model	(151)
NOD2 activation	Reduced SERT activity	IEC model	(152)
TLR2 deficiency	Decrement of gut 5-HT level	Tlr2 KO mice	(145)
TLR4 deficiency	Increased central 5-HT level	Tlr4 KO mice	(153)
TLR2/4 deficiency	Altered gut 5-HT receptors expression	Tlr2/4 DKO mice	(154, 155)
NODs deficiency	Altered gut 5-HT signaling	Nod DKO mice	(156)

Effects of activation of TLRs and NLRs on serotonergic system and effects of TLRs and NLRs deficiency on different in vivo and in vitro models.

### 3.1 Toll-Like Receptor 2

TLR2 is expressed in the GI tract in mononuclear cells of the lamina propria, goblet cells, and enterocytes (96), as well as neurons from the ENS (97). TLR2 is able to detect a broad range of MAMPs from several microorganisms, including Gram-positive bacteria through the formation of heterodimers with TLR1 (TLR2/1) and TLR6 (TLR2/6) (157), some fungi such as *Candida albicans* (158), viruses such as the hepatitis C virus (159), and some parasites such as *Trypanosoma cruzi* (160). At the CNS level, TLR2 is expressed in microglia, astrocytes, and oligodendrocytes (93). TLR2 in the brain mainly recognizes DAMPs as heat shock family proteins HSP60 and HSP70 (95) or high-mobility group box 1 proteins from dying tumor cells (HMGB1) (98), among others. However, the effect of TLR2 is not limited to immune responses. Previous results carried out in our laboratory have showed that TLR2 activation may modify the intestinal serotonergic system. TLR2 activation could decrease SERT activity due to a reduction in SERT protein expression, with cAMP/PKA and p38/MAPK intracellular pathways being implicated. Moreover, the expected increment of extracellular 5-HT will induce a negative feedback in TLR2 expression, supported by this cross-regulation between the TLR2 and serotonergic system (144). In fact, TLR2 and TLR4 activation may increase the production of IL-10 in the intestine (161), which in turn seems to modify SERT (162). In addition, TLR2 and TLR4 signaling seem to modulate GI motility mediated by 5-HT_2_, 5-HT_3_, 5-HT_4_, and 5-HT_7_ receptors (154). In line with these results, other researchers have found that TLR2 deficiency results in a decrement of gut 5-HT synthesis *in vivo* and that TLR2 activation upregulates the expression of TPH1 and 5-HT production in the gut (145). Serotonin-TLR2 relation is not limited to the GI tract, as previous data have highlighted that 5-HT_2B_ receptor activation downregulates TLR2 expression and TLR3-induced proinflammatory factors in the brain (150). Selective 5-HT_2A_ receptor antagonists activate glucocorticoid receptor nuclear translocation to upregulate TLR2 and TLR4 in response to microglial phagocytosis stimulation as a novel therapy in central pathologies such as Alzheimer’s disease (163).

### 3.2 Toll-Like Receptor 3

TLR3 is expressed in IECs, which mainly differentiates double-stranded RNA (dsRNA) from viruses. Surprisingly, TLR3 levels are age dependent because TLR3 expression increases after the suckling-to-weaning transition so as to give protection to the individuals against the virus as a rotavirus (164). In contrast, central TLR3 expression decreases during neurogenesis of the CNS in the embryo (165). TLR3 is also able to recognize endogenous mRNA as a DAMP from necrotic cells during intestinal inflammation (102). At the CNS level, TLR3 is expressed in a broad range of cells, including astrocytes, oligodendrocytes, and microglia cells (93), which is not surprising because viruses can easily reach the brain through other ways different from the BBB, such as neural pathways. Thus, TLR3 can detect dsRNA from the virus in the brain and trigger defense responses to protect the CNS against pathogens. Actually, TLR3 may protect the brain against some viruses such as the herpes simplex virus type 1 (HSV-1) (166). However, other microorganisms such as the Zika virus can activate TLR3 and induce an exacerbated inflammation and necrosis of the natural defenses of the brain, including the BBB (167). TLR3’s role in inflammatory responses may also be exacerbated by its potential pro-oxidant effect. In fact, TLR3 induces protein and lipid oxidation by reducing antioxidant enzymatic activity (168).

TLR3 activation is involved not only in inflammatory and oxidative damage–related responses but also in the modulation of the serotonergic system in the GI tract; TLR3 activation inhibits SERT activity and expression (146). In contrast, central TLR3 may have an opposite role because recent results have shown that TLR3 activation in a mice model with a brain infection increases SERT activity in astrocytes and therefore reduces extracellular 5-HT levels (147). In contrast to other TLRs, increased levels of 5-HT will not regulate TLR3 expression (146); meanwhile, other studies have reported that the activation of 5-HT_2B_ receptors may reduce TLR3 expression (150).

### 3.3 Toll-Like Receptor 4

TLR4 is one of the most studied PRRs, and its expression can be found in the apical membrane of IECs in the small intestine and in the basolateral membrane in the colon (110). In the brain, TLR4 is an important PRR in the glia because several researchers have reported its expression (105); meanwhile, TLR4 is expressed less often in astrocytes (106) and may be absent in oligodendrocytes (93). TLR4 recognizes the lipopolysaccharide (LPS), which is the fundamental component of Gram-negative bacteria walls. In this process, the myeloid differentiation factor 2 (MD-2) protein is critical because several studies have found that MD-2 deletion yields to the lack of detection of LPS by TLR4 (169), suggesting that MD-2 retains TLR4 in the cellular surface to detect LPS due to changes in TLR4 glycosylation (170). Due to the broad microorganisms that TLR4 can identify through LPS detection, TLR4 has been defined as a gate keeper of microbial homeostasis in the intestine, where it is involved in several defense mechanisms, including the zoonotic *Campylobacter* (171), *Helicobacter pylori* (172), or *Salmonella* (173). TLR4 could also have a regulator role in the serotonergic system. TLR4 modulates contractile response in the intestine and is mediated by serotonin receptors (154). TLR4 activation inhibits SERT activity through post-transcriptional mechanisms, leading to an increase in extracellular 5-HT (148). In addition, melatonin, a molecule linked with 5-HT synthesis, may modify intestinal microbiota composition through TLR4 signaling (174). At the CNS level, TLR4 participates in the detection of pathogens that cause meningitis, such as *Neisseria meningitidis* (175), where some DAMPs linked to brain damage mediate TLR4 signaling (176). Interestingly, recent results have pointed out that microbiota and TLR4 signaling are key players in Parkinson’s disease, one of the most important degenerative brain pathologies (177). In this context, previous studies have shown that the lack of TLR4 in the CNS leads to an increase in the central 5-HT level, suggesting the critical regulatory role of TLR4, not only in the GI tract but also in the central serotonergic system (153).

### 3.4 Toll-Like Receptor 5

TLR5 seems to be one of the most important TLRs in the GI tract because its expression and activity has been reported in all intestinal segments (122). In this context, TLR5 is expressed in the basolateral side of IECs from the colon, while in the small intestine, its expression is restricted to Paneth cells. TLR5 recognizes flagellin, a component that enables the motility of several bacteria. Several studies have indicated that flagellin origin is determinant in the defense response against bacteria because flagellin from pathogenic *Salmonella typhimurium* triggers a more exacerbated immune response than does flagellin from the nonpathogenic bacteria *E. coli* (178). In this context, TLR5 is a critical gatekeeper because it may control the intestinal microbiota composition by maintaining a physiological low grade of inflammation in the GI tract (179). Previous studies have extensively described TLR5 expression in microglia cells, where its function may be involved in the inflammatory diseases in the brain comprising bacteria that cause meningitis (180). However, TLR5 is not only involved in bacterial infection but can also be related with depression. Previous works have described how TLR3, TLR4, TLR5, TLR7, TLR8, and TLR9 mRNA expressions in peripheral blood mononuclear cells seem to be increased in patients with depression. The improvement of these patients through the use of selective serotonin reuptake inhibitors (SSRIs) indicates the implication not only of TLR5 but also other PRRs in the modulation of the serotonergic system in brain disorders (181).

### 3.5 Toll-Like Receptor 7 and Toll-Like Receptor 8

TLR7 and TLR8 are closely related PRRs expressed in endosomal membranes that can detect single-stranded RNA (ssRNA) (120). Previous works have described the lack of TLR7 expression in IECs, being mainly expressed in plasmacytoid dendritic cells (pDCs), in B cells at the lamina propria (122), and in the myenteric and submucous plexuses of murine intestine and human ileum (104). Meanwhile, TLR8 can be found in macrophages and monocyte-derived DCs (120). In both cases, it seems that TLR7 and TLR8 could have more importance in other organs, such as the respiratory system, than in the GI tract by recognizing respiratory viruses and triggering inflammatory responses (182). At the CNS level, TLR7 and TLR8 are mainly expressed in microglia cells. TLR7 acts by regulating the inflammation (183) and modulation of TLR9 expression (184); meanwhile, TLR8 is related with the attenuation of the outgrowth of neurons and the induction of apoptosis (185). In the GI tract, 5-HT can act by regulating TLR7 in DC through the 5-HT_2B_ receptor (150). Moreover, SSRIs seem to decrease the expression of both TLR7 and TLR8 in the CNS (181).

### 3.6 Toll-Like Receptor 9

TLR9 is included, together with TLR3, TLR7, and TLR8, in the group of TLRs that is classically expressed in membranes of intracellular organelles such as the endoplasmic reticulum, endosomes, and lysosomes. However, TLR9 can also be detected in endosomal locations (186). In the GI tract, TLR9 can be expressed in the apical and basolateral membrane of IECs to control homeostasis by means of various intracellular signaling (187). The intestinal map of TLRs describes TLR9 expression mainly in the lamina propria, and at low levels in GI epithelial cells (122). TLR9 recognizes unmethylated DNA found generally in microorganisms such as viruses and bacteria (127). However, TLR9 can also detect host DNA in aberrant locations, such as a DAMP of tissue damage (129), and it participates in the protection against GI damage and in GI repair (188). Moreover, TLR9 seems to act as an inhibitor of antimicrobial peptides in the intestine to avoid the colonization of pathogens (189). Because pathogen-free mice display a higher TLR9 expression in the intestine than germ-free mice do, it has been suggested that beneficial bacteria could modulate TLR9 expression in the GI tract (190). At the CNS level, TLR9 is expressed in microglia, neurons, and astrocytes (125), mediates immune responses related with brain infections, such as the herpes simplex virus (191), and attenuates brain injury (192). Little research has been carried out in the influence of TLR9 over the serotonergic system, and only a few works have indicated that SSRIs may modulate TLR9 mRNA expression in the peripheral blood mononuclear cells of depression patients (181) and will be implicated in the tryptophan catabolism (i.e., the main 5-HT resource) (193). In fact, preliminary data from our research group indicate that TLR9 could affect SERT activity and expression in an IECs model (194).

### 3.7 Toll-Like Receptor 10

TLR10 is the only PRR without known ligand specificity and biological function. Human TLR10 is encoded on chromosome 4 within the TLR2 gene cluster, together with TLR1, TLR2, and TLR6, suggesting a possible heterodimer TLR2/TLR10 (195). It has been described that TLR10 could act as an inhibitory receptor that essentially controls TLR2-driven signals (196). TLR10 is predominantly expressed in tissues rich in immune cells, such as the spleen, lymph node, thymus, tonsil, and lung (197). Genetic variations found in the TLR10 gene may cause a shift in the levels of pro- and anti-inflammatory responses and enhance the susceptibility to autoimmune diseases, cancers, and infections at the GI tract (198–200). Recently, TLR10 has been described in multiple mucosal sites, such as the small intestine, fallopian tubes, eyes, or stomach (198, 201, 202), suggesting a key role as a pathogen sensor in the mucosa. In the GI tract, TLR10 seems to be a chief component in the immune response to *Listeria monocytogenes* in IECs. In this context, previous studies have shown that *L. monocytogenes* affects SERT activity mediated by TLR10, which triggers the activation of a MyD88-dependent intracellular pathway (which may increase 5-HT uptake), and by a MyD88-independent downstream signaling (which may decrease 5-HT uptake), proving a deep involvement of TLRs in the serotonergic mechanism (131). At the CNS level, TLR10 could be critical for macrophage activity. In fact, microglial cells express TLR10, and this receptor inhibits M1 macrophage cytokines but promotes M2 cytokines, indicating that TLR10 may have a protective role in the brain (130).

### 3.8 NOD-Like Receptors

Like the TLRs, the NLRs are PRRs that detect both DAMPs and MAMPs triggering immune-related responses to protect the host. However, NLRs differentiate from TLRs with regard to the quality of being cytosolic receptors. NLRs can be classified into two big groups: the NLRC subfamily that encompasses the most popular, including NOD1, NOD2, and NLRC4, and the NLRP subfamily that includes up to 14 PRRs (203).

#### 3.8.1 *NOD1*

NOD1 is an intracellular PRR widely expressed in the organism with special relevance in the IECs and in the immune cells from lamina propria in the GI tract, where this PRR detects κ-d-glutamyl-meso-diaminopimelic acid (iE-DAP) from bacterial peptidoglycan, which can be found in most of the bacterial wall (136). NOD1 has been involved in the protection of the GI tract against pathogens such as *S. typhimurium* (204), *Citrobacter rodentium* (205), or *H. pylori* (206), among others. Previous works have described the expression of NOD1 in the CNS but at a lower level compared with TLRs (207), where one of the main functions is the protection against bacterial infections (208). Interestingly, NOD1 and NOD2 defense effects are only related with immunity because an elegant study has demonstrated that the lack of both receptors in mice leads to signs of stress-induced anxiety, cognitive impairment, and depression, together with increased GI permeability and altered serotonin signaling in the gut, suggesting that NOD1 and NOD2 are novel therapeutic targets for gut–brain axis disorders (156). Supporting these results, NOD1 activation may decrease SERT activity in IECs due to the diminishment of SERT expression. In turn, 5-HT levels seem also to upregulate NOD1 expression. However, NOD1 could also regulate other PRR expression such as TLR2 and TLR4 (151).

#### 3.8.2 *NOD2*

NOD2 is one of the most studied NLRs in the GI tract because polymorphisms in the gene that encodes NOD2 have been strongly associated with IBDs (209) and colorectal cancer (210). NOD2 is an intracellular PRR expressed in all IECs in the GI tract, which explains its implication in the protection of the intestine against the mentioned pathologies (211). NOD2 detects the bacterial peptidoglycan named muramyl dipeptide (MDP), which allows the identification of several pathogens, including *Yersinia* (212), *Campylobacter* (213), and *Listeria* (214). At the CNS level, NOD2 seems to play a similar role by detecting pathogens, triggering immune-related responses, and protecting the host (215). Like NOD1, NOD2 would be an important PRR in the gut–brain axis, especially because of its relation with the serotonergic system in both the CNS and the GI tract (156). In this sense, bacterial activation of NOD2 may decrease SERT activity and expression, thus leading to an increase in extracellular serotonin, and then serve as a negative feedback modulation of NOD2. In addition, NOD2 not only modulates the serotonergic system directly but also through its interdependence with TLR2 and TLR4 (152). In fact, the increase of extracellular 5-HT by NOD2 is not only for the downregulation of SERT but also for the increase of enterochromaffin cells that are responsible for 90% of the total 5-HT (216).

## 4 Intestinal Microbiota: Direct Effects on Serotonergic System

In recent years, intestinal microbiota involvement has gained high importance in numerous pathologies, including gut–brain disorders such as IBDs (217), depression (218), or Alzheimer’s disease (219). In this context, several studies have indicated that 5-HT and serotonergic system modulation by intestinal microbiota are critical in the maintenance of the gut–brain axis (220–222). Microbiota can produce tryptophan and tryptamine, directly affecting central 5-HT production (223). GF mice display a reduction in anxiety-like behavior compared with specific pathogen-free mice, showing a decreased expression of serotonin receptor 1A in the hippocampus (224). In the GI tract, microbiota increase the production of intestinal 5-HT by increasing TPH1 expression (225), and, more interestingly, microbiota can also synthesize 5-HT on their own (226). In agreement with this study, the alteration of microbiota composition and diversity seems to reduce host serotonin levels, increase tryptamine levels, and disrupt the GI immune system (227). However, it seems that microbiota not only influence 5-HT synthesis and SERT expression but also modulate the expression of some 5-HT receptors (228).

Some pathogenic bacteria such as *E. coli* can downregulate the activity and expression of SERT in the intestine (229), and an increase of extracellular 5-HT may induce an adherent-invasive *E. coli* colonization (230). Moreover, it has been described that *E. coli* can produce tryptophan, which will affect 5-HT production (231). Similarly, some beneficial bacteria such as *Lactobacillus* seem to degrade tryptophan, affecting central and intestinal 5-HT production (232). Several studies have shown that germ-free animals have a lower number of enterochromaffin cells compared to those with a standard microbiota (233). Specific pathogen-free mice display lower 5-HT levels (234), concluding that microbiota can regulate host 5-HT production not only at the intestinal level but also in the CNS (235). Apart from that, intestinal microbiota can produce tryptamine, the precursor of 5-HT, independently of the host (226), which introduces new strategies as to how microbiota will not only modify the intrinsic serotonergic system but also externally modify the levels of 5-HT in the host.

Moreover, intestinal microbiota can modify serotonergic systems by means of their metabolites and affect behavior through the modulation of 5-HT signaling (236). In this context, some metabolites, including the short-chain fatty acids (SCFAs), are a key component in this modulation and directly affect the gut–brain axis (237). SCFAs are metabolites from dietary fiber fermentation. They are characterized by having less than six carbon atoms, so they can easily cross membranes, including the BBB. Although studies on the physiological concentrations of SCFAs in the brain are scarce, the three main SCFAs—acetate, propionate, and butyrate—have been detected in cerebrospinal fluid (Human Metabolome Database. Available online at: http://www.hmdb.ca/). In fact, SCFAs could have a critical role in the maintenance and integrity of the BBB (238). SCFAs seem to regulate the expression levels of TPH1 in the intestine (239). In our lab, we have described that SCFAs can regulate intestinal SERT activity and expression (240). Similarly, other bacterial metabolites such as L-lactate seem to control the expression of 5-HT receptors 1B, 1D, and 4 in the CNS (241). In fact, there is a growing interest in the involvement of microbiota metabolites in the modulation of multiple neurochemical pathways through the highly interconnected gut–brain axis, which could be open novel approaches for gut–brain axis disorders (242).

## 5 Conclusions and Future Perspectives

**Figure 2:**
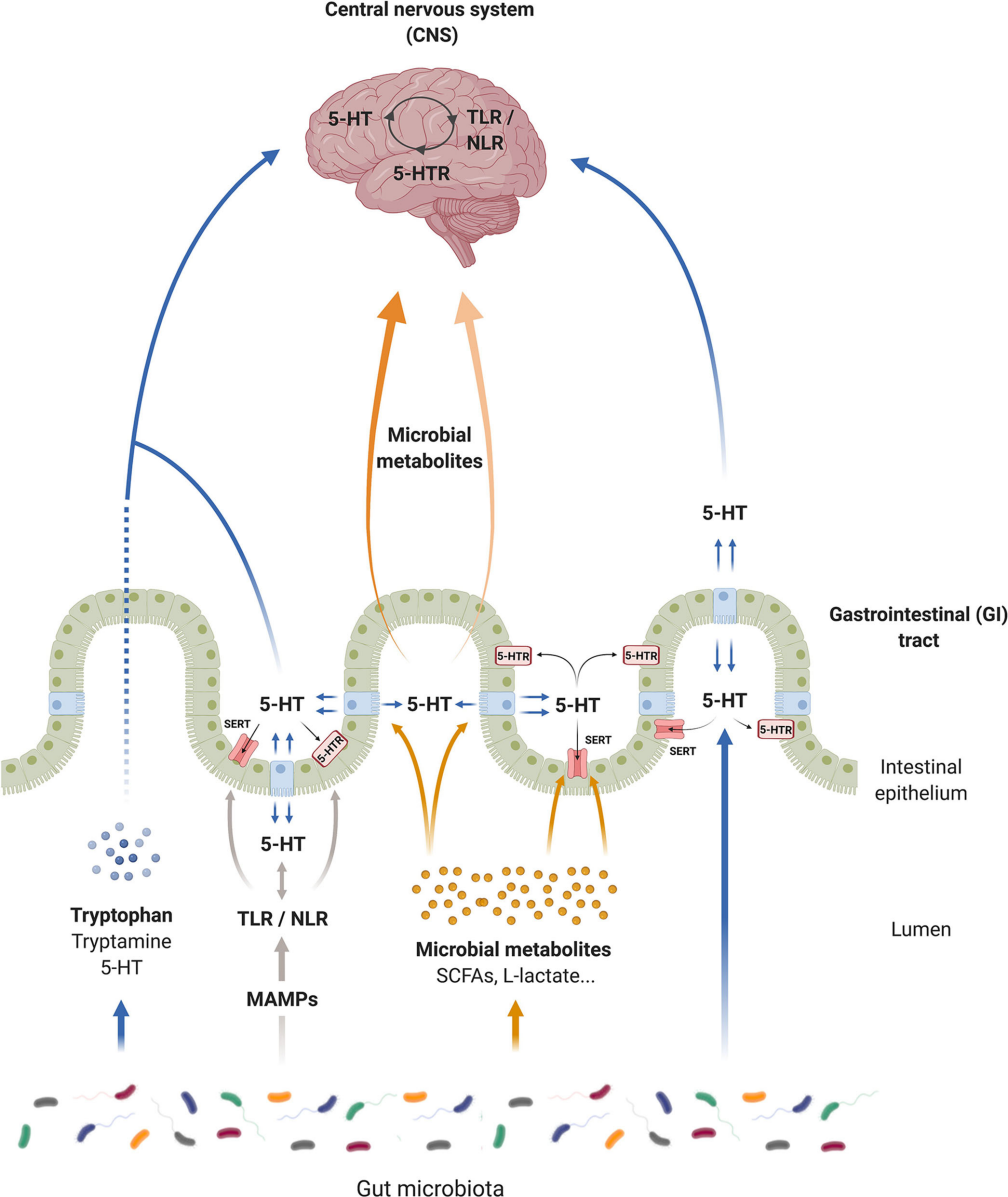
Serotonin (5-HT) communication pathways of the microbiota–gut–brain axis. Serotonin can modulate gastrointestinal (GI) and central nervous system (CNS) functions and is a key network for the gut–brain axis. Microorganisms produce tryptophan, and degrade tryptophan, affecting the central and intestinal 5-HT production. Intestinal microbiota modulate the synthesis of 5-HT and produce 5-HT independently of the host. Microbial associated molecular patterns from microorganisms (MAMPs) through toll-like receptors (TLRs) and nucleotide oligomerization domain (NOD)-like receptors (NLRs) affect directly the serotonergic system. TLR/NLR signaling seems to modulate the activity and the expression of serotonin transporter (SERT) and serotonin receptors (5-HTRs), as well as the 5-HT synthesis in the GI tract. However, this interconnection between TLRs/NLRs and serotonergic system exists in the CNS. In a feedback regulation, 5-HT affects pattern recognition receptor (PRR) expression. In addition, microbial metabolites, such as short chain fatty acids (SCFAs), can promote 5-HT synthesis by enterochromaffin (EC) cells and regulate SERT activity and expression. In the same way, these metabolites can migrate into the bloodstream to reach the brain, and some of them such as L-acetate can modulate the nervous serotonergic system, controlling the expression of 5-HT receptors.

The serotonergic system is the chief mechanism in the intestine that controls the GI tract (243) and the CNS physiology (244), with serotonin being one of the most important neurotransmitters in these organs. In addition, 5-HT modulates not only the GI tract and CNS functions, but also their interconnection (i.e., the gut–brain axis). In this context, numerous researchers have claimed that either 5-HT or tryptophan (main 5-HT resource) could be a key factor in gut–brain axis regulation (245) and that its imbalance could trigger pathologies in any of these organs (246). Interestingly, intestinal microbiota participate directly in 5-HT production, and by means of PRRs activation, microbiota can also affect SERT and regulate 5-HT levels. Moreover, changes in the extracellular 5-HT level may affect PRRs expression in a feedback regulation in order to maintain homeostasis (**Figure 2**).

Interestingly, various pathologies within the gut–brain axis that are apparently unrelated seem to have three common aspects: changes in intestinal microbiota, alterations of the intestinal serotonergic system, and dysfunction of the PRRs (**Table 4**). In the GI tract, IBDs, including CD and UC, are characterized by changes in the microbiota (345), alterations in the serotonergic system (346, 347), and dysfunction of the innate immune system, including TLRs (109) and NLRs (348). In recent years, novel IBD therapy has focused on treatment to reestablish these three components. Classical control of the intestinal microbiota has focused on antibiotics treatment. However, long-term use of antibiotics in IBDs seems not to resolve the inflammation and is associated with more harm than benefits (349). Fecal microbial transplantation is one of the most promising novel treatments in IBDs (350), together with the use of probiotics (351). In the last century, the use of anti-TNF has also been the most important drug intervention in IBD patients (352). However, this therapy may be insufficient, and novel studies have indicated that more treatments addressing innate immunity should be carried out. In this context, several studies have indicated that TLR (353) and NLR (354) modulation may help in the treatment of these chronic pathologies. Finally, serotonergic system modulation has been one of the main targets for IBD therapeutics in recent years. In this context, the inhibition of mucosal serotonin (355) or the use of inhibitors for SERT (356) may help in IBDs and thus be considered as a novel therapy for IBDs.

**Table 4:** Gut microbiota in intestinal disorders (IBD and IBS) and neurodegenerative pathologies and their interaction with serotonergic system.

Bacteria phyla	Bacteria family	Intestinal disorders	Central neurodegenerative pathologies	5-HT alterations	Therapeutic approach
Actinobacteria	Bifidobacteriaceae Coriobacteriaceae	Decreased in IBS (247, 248) Decreased in IBD (249, 250) Decreased in UC and CD (251) Increased in IBS (248) Decreased in IBD (249) Increased in UC and CD (252)	Increased in Parkinson’s disease (253–255) Decreased in Alzheimer’s disease (251) Decreased in autism spectrum disorder (256, 257) Increased in bipolar disorder (258) Decreased in Parkinson’s disease (259) Increased in bipolar disorder (258)	Increases TPH1 and decreases SERT expression (260) Increases 5-HT in CNS (261) Increases mucosal 5-HT, and expression of SERT; 5-HTR_2_ and 5-HTR_4_ (237) Bifidobacterium are decreased in SERT^-/-^ mouse (262) Correlated with increased levels of serotonin (263)	Microbial manipulation: prebiotic and probiotics in GI disorders (264–266) Probiotic supplementations in neurodegenerative disorders (267, 268); Microbial manipulation: microbiota transplant in GI disorders (269–272) Microbial manipulation: microbiota transplant in neurodegenerative disorders (273–276) Natural products in neurodegenerative disorders (277, 278) Natural products in GI disorders (279, 280) Dietary fibers in GI disorders (281) Nanotechnology and nanotheranostic approach in neurodegenerative disorders (282–284) Oxidized phospholipidis (285, 286) SCFAs in GI inflammatory disorders (287–289) CD36 in Alzheimer’s disease (290)
Firmicutes	Clostridiaceae	Decreased in IBS (291) Increased in IBD (292, 293) Increased in CD (293)	Increased in Parkinson’s disease (284, 294) Increased in autism spectrum disorder (256, 257) Decreased in bipolar disorder (258)	Increases mucosal 5-HT and EC cells and decreased SERT expression (295) Correlated with increased levels of serotonin (263) Increases SERT expression (296)	
	Lachnospiraceae	Decreased in IBS (291) Decreased in IBD (293, 297) Decreased in UC and CD (298) Not modified in CD (252) Increased in UC (252)	Decreased in Parkinson’s disease (259, 299, 300) Decreased in autism spectrum disorder (257, 301)	Correlated with increased levels of serotonin (263) Increased in Tph^-/-^ mice (6)	
	Ruminococcaceae	Reduced in IBD (293, 297) Increased in IBS (248, 302) Increased in UC (252) and in CD (293) Reduced in CD (252, 298)	Increased in Parkinson’s disease (259) Increased in Alzheimer’s disease (300) Decreased in autism spectrum disorder (301) Decreased in bipolar disorder (258)	Increases 5-HT levels (232) Correlated with levels of serotonin (303)	
	Veillonellaceae	Increased in IBS (304, 305) Increased in IBD (305, 306) Increased in UC and CD (252)	Decreased in autism spectrum disorder (256)	Correlated with increased levels of serotonin (307)	
	Lactobacillaceae	Increased in IBS (248) Increased in IBD (297) Decreased in IBD (*Lactobacillus*) (250) Increased in CD and reduced in UC (252)	Increased in Parkinson’s disease (253, 255, 308) Decreased in Alzheimer’s disease (251) Increased in Alzheimer’s disease (300) Increased in autism spectrum disorder (257)	Decreases TPH1, 5-THR_3_ and 5-HTR_4_ expression; and increases SERT expression (260) Increases 5-HT levels (309) Lactobacillus are increased in SERT^-/-^ mouse (262) Serotonin-producing bacterial strains (*Lactobacillus*) (310)	
	Enterococcaceae	Decreased in IBS (291) Increased in IBD (250, 311) Increased in CD (312) and UC (313)	Increased in Parkinson’s disease (299, 314) Increased in Alzheimer’s disease (251) Increased in autism spectrum disorder (257)	Enterococcus are increased in SERT^-/-^ mouse (262)	
	Staphylococcaceae	Increased in IBD (315)		Induces 5-HT release (316) 5-HT producers (317)	
	Listeriaceae	Increased in IBD (318)		SERT inhibition (132)	
Bacteriodetes	Bacteroidaceae	Increased in IBS (319) Reduced in IBD (297) Reduced in CD (252) and UC (320)	Increased in Parkinson’s disease (255, 259) Decreased in autism spectrum disorder (257, 321) Increased in state of anxiety (322)	Increased in Tph^-/-^ mice (6) Increases EC cells (323)	
	Tannerellaceae	Decreased in UC (252)		Increases 5-HT in hippocampus (324)	
	Rikenellaceae	Decreased in IBS (325) Decreased in IBD (297) Decreased in UC and CD (252)	Decreased in Parkinson’s disease (259) Increased in autism spectrum disorder (256, 257)	Correlated with levels of serotonin (303)	
	Prevotellaceae	Decreased in IBS (248) Increased in IBS (304) Increased in IBD (326) Increased in UC and CD (252)	Decreased in Parkinson’s disease (254, 327) Decreased in autism spectrum disorder (256, 321)		
Proteobacteria	Enterobacteriaceae	Increased in IBS (302) Increased in IBD (292, 306) Increased in UC and CD (252)	Increased in Parkinson’s disease (328) Increased in Alzheimer’s disease (300) Increased in autism spectrum disorder (257)	Decreases 5-HT and SERT protein (329) Increase 5-HT bioavailability (330) Increases EC cells (331) Serotonin-producing bacterial strains (*Escherichia coli K-12*) (332), (*Morganella morganii, Klebsiella pneumonia, Hafnia alvei*) (333)	
	Campylobacteraceae	Increased in IBD (334) Risk factor of IBS (335)		5-HT modulates *Campylobacter jejuni* physiology (336)	
	Helicobacteraceae	Reduced in IBD, UC and CD (337)	Increased in Alzheimer’s disease (251)	Increases 5-HT levels (338)	
Fusobacteria	Fusobacteriaceae	Increased in IBS (339) Increased in IBD (250, 306) Increased in CD (340) and UC (341)			
Verrucomicrobia	Akkermansiaceae	Reduced in IBD (297, 311) Reduced in UC (342)	Increased in Parkinson’s disease (254, 255, 327) Increased in autism spectrum disorder (256)	Increases SERT expression (296) Increases 5-HT in colon and hippocampus (8) Akkermansia are decreased in SERT^-/-^ mouse (262)	


The table summarizes the alterations of bacteria belonging to different bacterial families that are included in the six major phyla of the human gut microbiota (343, 344) in relation to inflammatory intestinal disorders (IBD, IBS, UC, and CD) and neurodegenerative pathologies (Alzheimer, Parkinson, etc.). Likewise, the table indicates the observed effects of the different bacteria on components of the serotonergic system or the bioavailability of serotonin. The last column lists some examples of therapeutic approaches related to the intestinal microbiota for the treatment of intestinal and neurodegenerative pathologies. IBD, inflammatory bowel disease; IBS, inflammatory bowel syndrome; UC, ulcerative colitis; CD, Crohn’s disease; CNS, central nervous system; GI, gastrointestinal; EC, enterochromaffin; TPH, tryptophan hydroxylase; SERT, serotonin transporter.

Irritable bowel syndrome (IBS) has been described as a gut–brain disorder, where the serotonergic system may be altered in both the intestine and the CNS (357). Interestingly, intestinal microbiota (358), as well as SCFAs and 5-HT, are altered in IBS patients (359). In addition, TLRs and NLRs play a chief role in the pathogenesis of IBS. In fact, several studies have indicated that some PRRs serve as predictive markers for the disease (360) because their expression is increased in the mucosa from IBS patients (361). Thus, it is not surprising that gut–brain axis modulation in IBS seems to be the most effective therapy in this pathology. Previous studies have shown that SERT regulation (362, 363) and the synthesis of 5-HT (364) may be important in the treatment of IBS. Moreover, serotonin therapy efficiency in IBS is improved through the modulation of microbiota (365, 366), and previous studies have suggested the immunomodulation of PRRs in this GI disease (367).

Surprisingly, disorders in the CNS may share the same alterations as the GI pathologies. In this context, serotonergic system alteration may be involved not only in depression and anxiety (368) but also in Parkinson’s disease (369), multiple sclerosis (370), amyotrophic lateral sclerosis (370), and autism spectrum disorder (371), among others. In fact, conventional treatment for CNS disorders, especially depression, has focused on the modulation of the serotonergic system by means of SSRIs (372). Important findings have been published in the last years regarding the changes of intestinal microbiota in the CNS pathologies. Recent data have shown that intestinal microbiota may be a critical susceptibility factor in the development of neurological disorders such as Alzheimer’s disease, autism spectrum disorder, multiple sclerosis, Parkinson’s disease (373), and depression in particular, where the modulation of the intestinal serotonin by the microbiota seems to be an important trigger (138, 374). In fact, certain bacteria families modulate tryptophan levels in blood plasma that can cross the BBB and thus influence the central serotonergic system (375). Within this context, novel therapies of brain pathologies, such as Alzheimer’s disease, are focused on the modulation of intestinal microbiota to prevent and ameliorate the development of the pathology (376). These new studies have shown that the balance of the gut–brain axis is critical in preventing the development of GI and brain disorders mediated by 5-HT (377). Innate immune receptors, including TLRs and NLRs, could also be a key component in the correct function of the microbiota–gut–brain axis. Previous works have shown that TLR modulation by means of intestinal microbiota may be a critical factor in the development of brain disorders such as Parkinson’s disease (177, 378); in addition, NLRs may be involved in CNS inflammation and neurodegenerative diseases (379). New therapeutics have shown that the use of antidepressants may improve the negative regulation of PRRs in some CNS disorders such as depression (380), especially for TLR4 (381).

Based on the numerous studies focusing on the gut–brain axis, it is clear that the balance of this bidirectional communication may be important in the prevention of GI and CNS disorders, where the intermodulation of the microbiome, serotonergic system, and innate immunity is critical in maintaining homeostasis. However, more studies are needed to understand the implication of these elements, as well as their modulation as novel therapeutic targets, for the GI and CNS pathologies.

## Author’s Note

In memoriam: This paper is dedicated to the memory of Professor Ana Isabel Alcalde, a brilliant and enthusiastic scientist, professor, and colleague, as well as our director and mentor, who dedicated her last 20 years to the study of the serotonergic system.

## Author Contributions

Conceptualization: ElL and BB. Investigation: ElL and BB. Writing—Original Draft Preparation: ElL. Writing—Review and Editing: JM and EvL. Supervision: EvL. All authors contributed to the article and approved the submitted version.

## Funding

This work was funded by grants from the Foundation for the Study of Inflammatory Bowel Diseases in Aragón (ARAINF 2012/0567) and the Aragón Regional Government (A20_20 R).

## Conflict of Interest

The authors declare that the research was conducted in the absence of any commercial or financial relationships that could be construed as a potential conflict of interest.

## Publisher’s Note

All claims expressed in this article are solely those of the authors and do not necessarily represent those of their affiliated organizations, or those of the publisher, the editors and the reviewers. Any product that may be evaluated in this article, or claim that may be made by its manufacturer, is not guaranteed or endorsed by the publisher.
